# Organisms Causing Postoperative Implant Infection in Orthopedic Patients Presenting at a Tertiary Care Hospital

**DOI:** 10.7759/cureus.70821

**Published:** 2024-10-04

**Authors:** Raja Muhammad Mussab, Sharjeel Khan, Saad Zulfiqar Bubak, Abdulaziz Madni, Usman Ishaq, Shehzadi Rimsha, Shehanshah Muhammed Arqam, Hadia Javed

**Affiliations:** 1 Trauma and Orthopaedics, Jinnah Postgraduate Medical Centre, Karachi, PAK; 2 Trauma and Orthopaedics, Russells Hall Hospital, Dudley, GBR; 3 General Surgery, Sindh Government Hospital New Karachi, Karachi, PAK; 4 Internal Medicine, Civil Hospital Karachi, Dow University of Health Sciences, Karachi, PAK

**Keywords:** klebsiella species, orthopedic implant infections, orthopedics and trauma, ortho surgery, postoperative infections, staphylococcus aureus, surgical site infections

## Abstract

Introduction

Infections pose a significant challenge in orthopedics related to implant failure. In orthopedic surgeries, surgical site infections (SSIs) extend the patient’s hospital stay by an average of two weeks and also increase morbidity, double hospitalization rates, and triple the financial burden on the patient. This study aims to determine the common organism in patients with postoperative implant infections presenting at a tertiary care hospital.

Methods

A cross-sectional study was conducted in the Department of Orthopedic Surgery at JPMC Karachi over a six-month period, from November 24, 2022, to May 25, 2023. All patients of both genders aged 18-65 years presenting with postoperative implant infection within six hours of the development of symptoms were enrolled. Baseline demographic details and clinical histories were recorded at the time of presentation. A swab for culture and sensitivity was taken from the implant site using a sterile swab stick in patients with confirmed or suspected infections.

Results

Of 196 patients, the mean age of the patients was 48.47±5.19 years. Gender distribution showed that 49% of patients were females and 51% were males. The mean duration of surgery was 1.71±0.49 hours (100 minutes approximately); *Klebsiella* species 26% and *Staphylococcus aureus* 25.5% were the most common organisms isolated from infected surgical wounds, followed by Pseudomonas species in 32 patients (16.3%), *Acinetobacter* species in 27 patients (13.8%), *Escherichia Coli* in 23 patients (11.7%), and coagulase-negative *Staphylococcus* in 13 patients (6.6%).

Conclusion

In our cohort, *Staphylococcus* species and *Klebsiella* species were the most common pathogens isolated from postoperative implant infections. The rise in *Klebsiella* species suggests that changes in prophylactic antibiotic practices may have contributed to this trend. Therefore, there is an urgent need to reassess current prophylactic strategies in light of the increasing incidence of infections caused by gram-negative bacteria.

## Introduction

Infections that occur at the site of an invasive procedure are commonly referred to as surgical site infections (SSIs) [1]. In clinical practice, it is considered if the infection occurred within 30 days after the surgery or within one year if the implant remains in the body and affects the scar or the deeper tissues at the site of the surgery [2]. Surgery that involves orthopedic implant placement is frequently associated with chronic infections because of biofilm development, which complicates the eradication of these infections. The overall infection rate after orthopedic surgery with an implant is estimated to be around 5% [3]. Infections are a major problem in orthopedics in relation to implant failure.

SSIs can develop as deep or superficial incisional infections, or they may involve body spaces or organs. These infections are most common in hospitals and are associated with considerable morbidity, with more than a third of postoperative mortality caused by SSIs [4,5]. The presence of an SSI can double a patient’s hospital stay and, therefore, costs [5]. Several factors increase the risk of infection, including advanced age, existing infections elsewhere in the body, the use of systemic steroids, smoking, alcohol consumption, and the transfusion of certain blood products [6]. The severity of an infection depends on the pathogen’s virulence, the number of microorganisms involved, the location of the infection, the patient’s immune response, and any factors that may cause immunosuppression [7].

In orthopedic procedures, surgical instruments like screws, plates, and external fixation devices can contribute to the risk of infection [8]. The literature reports the presence of bacteria, such as *Staphylococcus aureus*, on items like pens, keyboards, stethoscopes, medical gowns, tourniquets, and sphygmomanometers, which increases the risk [9]. Common signs of an infected wound include pain and fever [10]. The likelihood of an SSI is influenced by the contamination of the wound site and the pathogenicity of microorganisms, which are counterbalanced by the host's immune response [11,12]. The organisms responsible for SSIs typically originate from the patient’s endogenous environment, such as their skin or an opened viscus. However, exogenous causes can also occur when the surgical instruments or the operating room environment introduce contaminants during the procedure [2,13]. The management of SSIs depends on identifying their susceptibility to antibiotics. Extended-spectrum *β-lactamases* are enzymes secreted by gram-negative bacilli, which have resistance to penicillin, cephalosporins, and monobactams. These enzymes are frequently found in *Enterobacteriaceae* and *Pseudomonas aeruginosa *[14]. Hence, it is important to know the type of pathogen commonly causing SSIs to develop a treatment plan. Mundhada and Tenpe have found Staphylococcus aureus as the most common organism isolated among patients with SSIs [15]. However, the focus was on patients from obstetrics & gynecology, general surgery, and orthopedic department. Similarly, most of the available literature focuses on postoperative SSIs in general patients. Hence, this study focuses on orthopedic patients and aims to determine the common organism in patients with SSIs after implant placement.

## Materials and methods

This study was a cross-sectional study and was conducted in the Department of Orthopedic Surgery at JPMC Karachi over a six-month period, from November 24, 2022, to May 25, 2023.

The study commenced after receiving approval from the ethical review committee of the institute and the College of Physicians & Surgeons of Pakistan. Patients meeting the inclusion criteria were recruited from the emergency department and the Orthopedic outpatient department. The inclusion criteria for the study were patients of both genders, aged 18 to 65 years, who presented with postoperative implant infections and sought medical attention within six hours of the onset of symptoms. Exclusion criteria included a history of pre-operative infected wounds, patients undergoing emergency surgery, and those with pre-existing cardiac, pulmonary, chronic liver disease, or renal disease, as well as immunocompromised patients. Informed consent was obtained from each patient prior to enrollment, with the details of the study thoroughly explained to them.

The sample size for the study was calculated using the World Health Organization sample size calculator, based on a frequency of 15% for *Pseudomonas *species in patients with implant infections, an error margin of 5%, and a 95% confidence interval. The calculation resulted in a required sample size of 196 participants. The sampling technique employed was nonprobability consecutive sampling [13].

Baseline demographic details and clinical histories were recorded at the time of presentation. All patients were evaluated for postoperative implant infections. For patients with confirmed or suspected infections, the wound was opened under aseptic conditions in the operating theatre, and a swab for culture and sensitivity was taken from the implant site using a sterile swab stick, which was then placed in a sterile test tube. The identification of organisms was conducted if there is the presence of one or more of the following signs and symptoms, i.e., pain and tenderness, localized swelling, and fever (temperature 100°F) along with the positive culture of tissue or fluid taken from the superficial wound. The study collected various variables, including age, gender, place of residence, family monthly income, height, weight, body mass index (BMI), mode of injury, duration of surgery, smoking status, hypertension, diabetes, American Society of Anesthesiologists (ASA) class, and isolated organisms, using a predesigned questionnaire.

For data analysis, IBM SPSS Statistics for Windows, Version 26 (Released 2019; IBM Corp., Armonk, New York, United States) was utilized. The normality of quantitative variables, such as age, height, weight, BMI, family monthly income, and duration of surgery, was assessed using the Shapiro-Wilk test. Depending on normality, the data were reported as either mean ± standard deviation (SD) or median (interquartile range). Qualitative variables, including gender, place of residence, mode of injury, smoking status, hypertension, diabetes, ASA class, and isolated organisms, were reported as frequency and percentage. Effect modifiers such as age, gender, place of residence, family monthly income, BMI, mode of injury, duration of surgery, smoking status, hypertension, diabetes, and ASA class were controlled through stratification. Poststratification, a chi-square or Fischer exact test was conducted, with a p-value of ≤0.05 considered statistically significant.

## Results

A total of 196 patients were included in the study. The patient population presents with a mean age of 48.47 years, ranging from 45 to 65 years, reflecting a midlife demographic. The average weight of the patients is 60.14 kg, with a variation from 53 to 66 kg, while their mean height stands at 1.53 meters, ranging between 1.50 and 1.63 meters. The BMI for these individuals averages 27.26 kg/m², indicating a generally overweight population with a span from 18.70 to 33.00 kg/m². The surgical procedures undertaken in this group lasted, on average, 1.71 hours, varying between 1.00 and 3.00 hours. Additionally, the average family income of the patients is recorded at 59,280 rupees, with incomes spanning from 34,000 to 95,000 rupees (Table 1).

**Table 1:** Demographic and Clinical Characteristics of Patients BMI: Body mass index

Characteristics	Mean ± SD	Minimum	Maximum
Age of the patients (years)	48.47 ± 5.19	45	65
Weight of the patients (kg)	60.14 ± 5.11	53	66
Height of the patients (m)	1.53 ± 0.06	1.50	1.63
BMI of the patients (kg/m²)	27.26 ± 4.94	18.70	33.00
Duration of the surgery (hours)	1.71 ± 0.49	1.00	3.00
Family income of the patients (rupees)	59,280 ± 13,162	34,000	95,000
	Frequency (%)		
Residence status			
Rural	67 (34.20%)		
Urban	129 (65.80%)		

**Figure 1:**
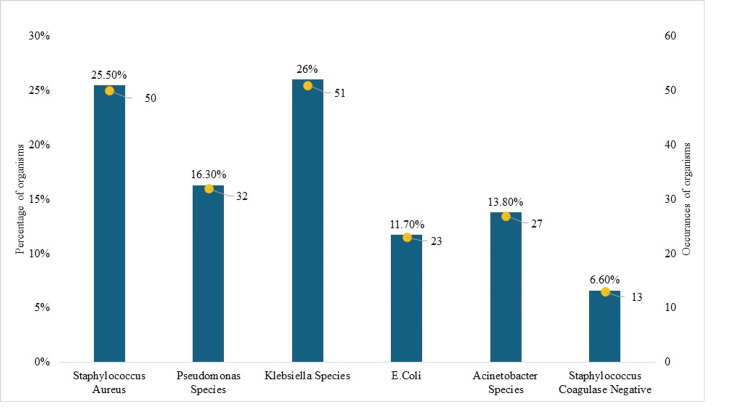
Frequency and Percentage of Common Organisms Isolated in Postoperative Implant Infection Patients

Figure 1 presents the frequency and percentage distribution of organisms isolated in patients with postoperative implant infections, which directly addresses the core research objective. Among the 196 cases analyzed, *Staphylococcus aureus* was the most prevalent, accounting for 50 cases or 25.5% of the total. This highlights the significant role of *Staphylococcus *in postoperative implant infections, underlining its importance in clinical settings and potential need for targeted interventions.

*Klebsiella* species were close to *Staphylococcus aureus*, with 51 cases or 26% of the infections, suggesting its equally high rate in this patient group. *Pseudomonas *species were also identified, with 32 cases or 16% of the total isolates.

*E. coli *was isolated in 23 cases and constituted 11.7% of the infections, whereas *Acinetobacter *species were isolated in 27 cases, which constituted 13.8% of the cases. While these organisms were not as frequent as *Staphylococcus *and *Klebsiella*, their frequency means that they also make a significant contribution to the infection load. In addition, coagulase-negative *Staphylococcus *was identified in 13 cases, or 6.6% of the infections. Although less frequently found than the other organisms, their identification is crucial to understand the broader spectrum of pathogens that can complicate postoperative recovery. Thus, various organisms cause postoperative implant infection; however, *Staphylococcus *and *Klebsiella *are the most common.

When examining the distribution of demographic and clinical characteristics (Table 2), it is noted that a significant majority, 65.80%, of patients reside in urban areas, while 34.20% come from rural settings. The predominant mode of injury among these patients is road traffic accidents, accounting for 61.70% of cases, with falls contributing to 38.30%. When considering lifestyle factors, 34.70% of the patients are smokers, whereas 65.30% do not smoke. In terms of comorbidities, 38.80% of patients have hypertension, and 44.40% have diabetes. The majority of patients, 78.60%, are classified as ASA II, indicating mild systemic disease, while the remaining 21.40% are categorized as ASA I, representing a generally healthy status.

**Table 2:** Distribution of Patient Clinical Characteristics and Medical History RTAs: Road traffic accidents; ASA: American Society of Anesthesiologists

Variables	Frequency (n)	Percentage (%)
Mode of injury		
RTA	121	61.70%
Fall	75	38.30%
Smoking		
Yes	68	34.70%
No	128	65.30%
Hypertension		
Yes	76	38.80%
No	120	61.20%
Diabetes		
Yes	87	44.40%
No	109	55.60%
ASA		
I	42	21.40%
II	154	78.60%

A detailed comparison of the common isolated organisms in postoperative implant infections across various baseline characteristics is also carried out. This comparison facilitates understanding how different factors such as age, gender, BMI, family income, and others might influence the prevalence of specific bacterial infections in these patients (Table 3).

**Table 3:** Combined Comparison of Common Isolated Organisms with Demographic and Clinical Characteristics (n=196) BMI: Body mass index; RTAs: road traffic accidents; ASA: American Society of Anesthesiologists The chi-square test was used with significance level p≤0.05

Characteristic	Staph. aureus	Pseudomonas Sp.	Klebsiella Sp.	E. coli	Acinetobacter Sp.	Staph. Coagulase Negative	p-value
Age (years)							
≤45	10 (15.4%)	11 (16.9%)	15 (23.1%)	8 (12.3%)	17 (26.2%)	4 (6.2%)	0.010
>45	40 (30.5%)	21 (16.0%)	36 (27.5%)	15 (11.5%)	10 (7.6%)	9 (6.9%)	
Gender							
Male	24 (24.0%)	15 (15.0%)	33 (33.0%)	10 (10.0%)	12 (12.0%)	6 (6.0%)	0.376
Female	26 (27.1%)	17 (17.7%)	18 (18.8%)	13 (13.5%)	7 (7.3%)	7 (7.3%)	
BMI (kg/m²)							
≤27.5	25 (28.7%)	11 (12.6%)	24 (27.6%)	9 (10.3%)	12 (13.8%)	6 (6.9%)	0.798
>27.5	25 (22.9%)	21 (19.3%)	27 (24.8%)	14 (12.8%)	15 (13.8%)	7 (6.4%)	
Family income (rupees)							
≤60,000	36 (27.3%)	22 (16.7%)	31 (23.5%)	15 (11.4%)	16 (12.1%)	12 (9.1%)	0.301
>60,000	14 (21.9%)	10 (15.6%)	20 (31.3%)	8 (12.5%)	11 (17.2%)	1 (1.6%)	
Duration of surgery (hours)							
≤1.5	22 (25.3%)	9 (10.3%)	22 (25.3%)	11 (12.6%)	14 (16.1%)	9 (10.3%)	0.190
>1.5	28 (25.7%)	23 (21.1%)	29 (26.6%)	12 (11.0%)	13 (11.9%)	4 (3.7%)	
Residence							
Rural	15 (22.4%)	18 (26.9%)	16 (23.9%)	8 (11.9%)	8 (11.9%)	2 (3.0%)	0.081
Urban	35 (27.1%)	14 (10.9%)	35 (27.1%)	15 (11.6%)	19 (14.7%)	11 (8.5%)	
Mode of injury							
RTA	30 (24.8%)	19 (15.7%)	34 (28.1%)	12 (9.9%)	18 (14.9%)	8 (6.6%)	0.872
Fall	20 (26.7%)	13 (17.3%)	17 (22.7%)	11 (14.7%)	9 (12.0%)	5 (6.7%)	
Smoking status							
Yes	16 (23.5%)	7 (10.3%)	19 (27.9%)	8 (11.8%)	12 (17.6%)	6 (8.8%)	0.478
No	34 (26.6%)	25 (19.5%)	32 (25.0%)	15 (11.7%)	15 (11.7%)	7 (5.5%)	
Hypertension							
Yes	18 (23.7%)	14 (18.4%)	18 (23.7%)	11 (14.5%)	11 (14.5%)	4 (5.3%)	0.857
No	32 (26.7%)	18 (15.0%)	33 (27.5%)	12 (10.0%)	16 (13.3%)	9 (7.6%)	
Diabetes mellitus							
Yes	24 (27.6%)	14 (16.1%)	23 (26.4%)	11 (12.6%)	11 (12.6%)	4 (4.6%)	0.912
No	26 (23.9%)	18 (16.5%)	28 (25.7%)	12 (11.0%)	16 (14.7%)	9 (8.3%)	
ASA							
I	10 (23.8%)	6 (14.3%)	10 (23.8%)	8 (19.0%)	6 (14.3%)	2 (4.8%)	0.696
II	40 (26.0%)	26 (16.9%)	41 (26.6%)	15 (9.7%)	21 (13.6%)	11 (7.1%)	

The comparison between different age groups highlights that younger patients (≤45 years) have a higher proportion of *Acinetobacter *sp. (26.2%) compared to older patients (>45 years), who have a higher proportion of *Staphylococcus aureus* (30.5%) and *Klebsiella *species (27.5%). The difference in the distribution of organisms between the age groups is statistically significant (p=0.010), suggesting that age may influence the prevalence of certain organisms. On the other hand, with respect to gender, BMI, ASA, family income, duration of surgery, residence, mode of Injury, smoking status, hypertension, and diabetes mellitus, the distribution of organisms shows some variation, though not statistically significant (Table 3). These findings suggest that while certain demographic and clinical factors might influence the prevalence of specific organisms, age is a more definitive factor in this context.

## Discussion

Our study identifies *Klebsiella *species and *Staphylococcus aureus* as the most prevalent pathogens causing SSIs in orthopedic implant surgeries. These results are similar to those in previous studies, which have identified *Staphylococcus aureus* as the leading pathogen responsible for implant-related infections [13,15-17]. However, data derived from the current study demonstrate a change in the infection pattern, with SSIs increasingly being attributed to *Klebsiella *species. This shift could be due to alterations in the prophylactic antibiotic regimes. The last few years have seen amoxicillin-clavulanic acid emerge as the first-line prophylactic antibiotic [18]. Amoxicillin-clavulanic acid is more potent against gram-positive bacteria such as *Staphylococcus aureus*, which used to be the most predominant pathogen in the past. As a result, this change could have led to an increase in infection rates by gram-negative bacteria like *Klebsiella *[19], which are less sensitive to amoxicillin-clavulanic acid.

Similar to our findings, Tandon et al. found that gram-negative *Enterobacteriaceae*, such as *Klebsiella *and *Escherichia coli*, were accountable for 62.79% of SSIs in clean orthopedic implant surgeries [20]. Agrawal et al. [21] and Lalremruata et al. [22] similarly reported that gram-negative bacteria were more common in infection profiles, contributing to 74.8% and 66.94% of isolates, respectively. This trend is also supported by Okoro’s study in which *Escherichia coli *was found to be the leading pathogen in orthopedic SSIs [23].

The observed prevalence of *Staphylococcus aureus* (25.5%) and *Klebsiella *species (26%) in our study corroborates earlier findings by Mukherjee et al., who reported similar percentages for *Staphylococcus aureus* (39%) and *Klebsiella *species (17%) [24]. Additionally, Jagiasi et al. reported *Staphylococcus aureus *as the most common pathogen in their cohort, followed by *Acinetobacter* and *Pseudomonas *[25]. This similarity across studies indicates the persistence of *Staphylococcus aureus* as a major pathogen while highlighting the increasing role of *Klebsiella *species.

Our study did not assess antibiotic susceptibility patterns directly. However, previous research indicates that *Escherichia coli*, *Klebsiella *spp., and *Staphylococcus aureus* are generally sensitive to imipenem and gentamicin [26]. Specifically, imipenem has been identified as highly effective against these bacteria [13,17]. Also, *Klebsiella *is sensitive to levofloxacin and cefuroxime, and on the other hand, *Staphylococcus aureus* is sensitive to levofloxacin and ciprofloxacin [27]. Conversely, all the isolated bacteria in our study are resistant to amoxicillin-clavulanic acid, probably because of its prolonged use. This might be an indication that there is a need for the change of our prophylactic antibiotic regimen to another one. Regarding the resistance patterns and new generation of gram-negative bacteria, it is time to reconsider our antibiotic policies and look for agents that are more effective against the modern spectrum of pathogens.

Some of the methodological implications of this research design include the inability to establish causality and the inability to monitor the long-term trends in infection rates. Thus, further research on this subject should be focused on longitudinal research that will assess the rates of infection and resistance patterns within a longer time period. The integration of antibiotic susceptibility testing in these studies would be valuable in obtaining information that could help in the modification of treatment plans.

## Conclusions

The most common pathogens isolated from postoperative implant infections were *Staphylococcus aureus *and *Klebsiella *species, with *Staphylococcus aureus* responsible for 25.5% and Klebsiella for 26% of the cases. The observed increase in *Klebsiella *species along with resistance to amoxicillin-clavulanic acid may be attributed to alterations in the prophylactic antibiotic usage. Consequently, it is imperative to reassess current prophylactic strategies, particularly given the rising incidence of infections caused by gram-negative bacteria. Clinicians should consider antibiotics effective against gram-negative organisms, such as imipenem, gentamycin, levofloxacin, and cefuroxime. The knowledge of the local resistance patterns and periodic antibiotic resistance monitoring could help control the infections and improve the patient’s status.
